# Magnetic Force-Driven Graphene Patterns to Direct Synaptogenesis of Human Neuronal Cells

**DOI:** 10.3390/ma10101151

**Published:** 2017-10-02

**Authors:** Kyung-Joon Min, Tae-Hyung Kim, Jeong-Woo Choi

**Affiliations:** 1 Department of Biomedical Engineering, Sogang University, 35 Baekbeom-ro (Sinsu-dong), Mapo-gu, Seoul 121-742, Korea; kjdmin@gmail.com; 2 School of Integrative Engineering, Chung-Ang University, Heukseok-dong, Dongjak-gu, Seoul 156-756, Korea; 3 Department of Chemical and Biomolecular Engineering, Sogang University, 35 Baekbeom-ro (Sinsu-dong), Mapo-gu, Seoul 121-742, Korea

**Keywords:** GO-encapsulated magnetic nanoparticles, GO hybrid pattern, human neuronal cell, synapse, axonal growth guidance

## Abstract

Precise control of axonal growth and synaptic junction formation are incredibly important to repair and/or to mimic human neuronal network. Here, we report a graphene oxide (GO)-based hybrid patterns that were proven to be excellent for guiding axonal growth and its consequent synapse formation of human neural cells. Unlike the previous method that utilized micro-contacting printing technique to generate GO patterns, here, GO-encapsulated magnetic nanoparticles were first synthesized and utilized as core materials wherein the external magnetic force facilitated the transfer of GO film to the desired substrate. Owing to the intrinsic property of GO that provides stable cell attachment and growth for long-term culture, human neuronal cells could be effectively patterned on the biocompatible polymer substrates with different pattern sizes. By using magnetic force-driven GO hybrid patterns, we demonstrated that accumulation and expression level of Synaptophysin of neurons could be effectively controlled with varying sizes of each pattern. The synaptic network between each neuron could be precisely controlled and matched by guiding axonal direction. This work provides treatment and modeling of brain diseases and spinal cord injuries.

## 1. Introduction

Recently, neural engineering or brain engineering has emerged in the field of regenerative medicine owing to its potential to precisely mimic human neural network (e.g., brain, spinal cord, neuromuscular junction) in vitro and to develop a new type of tool to treat the patients suffering various neurological diseases/disorders [1,2,3,4,5,6,7,8,9,10,11]. A number of approaches have been reported to be successful, including the generation of multiple types of neurons (e.g., dopaminergic, GABAergic, glutamatergic), guiding axons of neurons, construction of neural circuit, development of neural network-on-a-chip and the formation of brain organoids [12,13,14,15,16,17,18,19,20,21,22]. Among them, one of the promising area to be investigated is the guidance of axonal growth and its corresponding synapse formation, since the synaptic junctions are the key parts of the complex neuronal network system wherein signal transduction between each neuron actually occurs [23,24].

Interestingly, a rising two dimensional material, graphene (G) or graphene oxide (GO), has been known to possess distinct ability to control neural cell behaviors such as cell growth, adhesion, differentiation and migration [25,26,27,28,29,30,31]. Of these characteristics, one of the interesting feature of G/GO is the ability to enhance neurogenesis/oligodendrogenesis of neural stem cells (NSCs), with guided alignment of these differentiated neurons toward specific direction [32,33,34]. This was found to be contributed by the unique physicochemical characteristics of G/GO, which enhance the neural cell adhesion on the different types of substrates and also facilitate absorption/repulsion of growth factors/proteins on its surface [35,36,37,38,39]. Interestingly, both G/GO were reported to be patterned as stable and biocompatible cell-supporting materials. Thus, the patterning of graphene derivatives will be a promising candidate to control neuronal cell adhesion and to direct axonal alignment, which will ultimately be useful to guide synaptogenesis and synaptic junction formation of different neurons.

**Figure 1:**
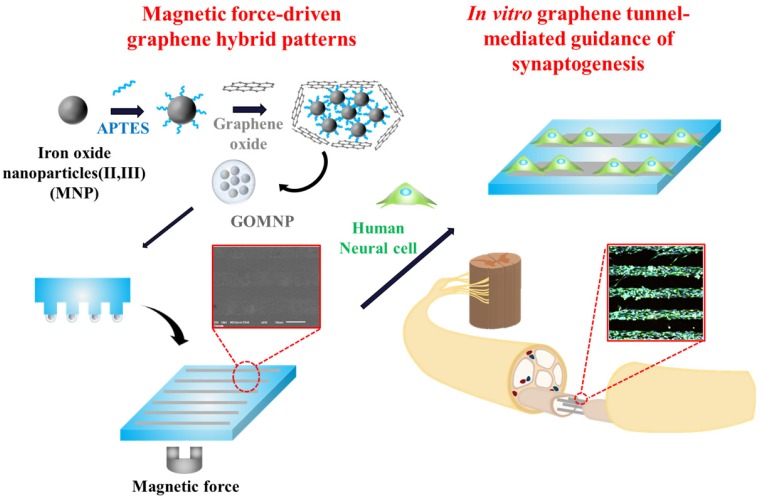
Schematic diagram illustrating synthesis of GO-encapsulated magnetic nanoparticles and conformation of GO hybrid pattern. By using this method, synaptogenesis guidance can be controlled.

Hence, in this study, we report a graphene pattern-mediated guidance of axonal growth and controlled synaptic junction formation of different neurons. GO-encapsulated magnetic nanoparticles (GOMNPs) were synthesized and then, transferred to the flexible polymer substrates to generate desired patterns. Owing to the magnetic force applied to the GOMNPs during the patterning process, we first demonstrate that the GO hybrid patterns could be easily and rapidly (<5 min) generated on the desired surface with high reproducibility, which is better than the patterns generated by conventional micro-contact printing method. To realize full potential of the magnetic force-driven GO hybrid patterns, the generated patterns were used as a template to control cell morphologies, axonal alignment and synaptophysin expression of human neuronal cells. The work was further extended to show the ability of these patterns to guide and regulate synaptic junction formation of neuronal cells growing from two different directions, which will be critical for mimicking complex human neuronal network and for treating brain/spinal cord injuries (Figure 1).

## 2. Results and Discussion

### 2.1. Generation of GO Hybrid Patterns with Different Geometries

**Figure 2:**
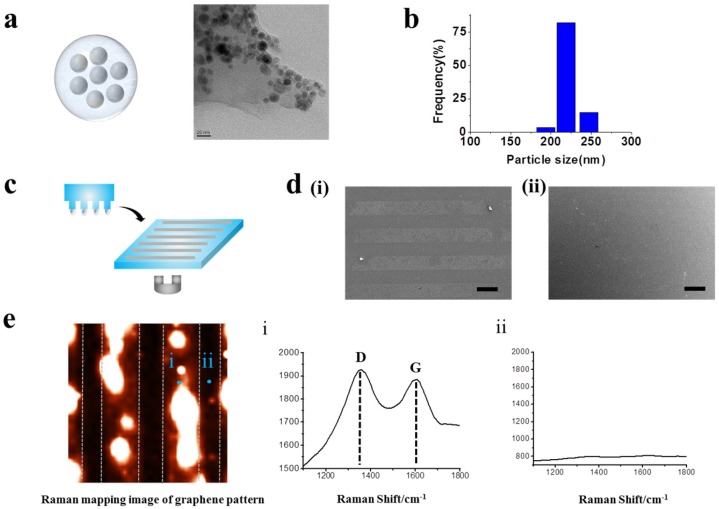
Pattering GO-encapsulated nanoparticles. (**a**) Schematic diagram and TEM images of synthesized GOMNPs. (**b**) DLS data of synthesized GOMNPs. (**c**) Schematic diagram illustrating patterning with and without magnetic force. (**d**) SEM images of patterns generated on polydimethylsiloxane (PDMS) substrate. Patterned with magnetic force (**i**) and without magnetic force (**ii**) (Scale bar: 100 μm). (**e**) Raman imaging of GO hybrid line pattern generated on PDMS. The *X* and *Y* axes were 100 μm × 100 μm. Raman spectra obtained from the spot (**i**) and (**ii**) from the Raman mapping image that proves the presence and absence of GO based on strong Raman peak.

In order to generate highly stable GO hybrid patterns with less fabrication time, we applied external magnetic force to facilitate transfer of GO layer to the desired substrate during micro-contact printing (MCP) process. MCP is a simple and effective method to create micro-sized patterns over large surface area [34,40,41,42,43,44]; however, in the case of GO patterning using MCP technique, owing to the nature of GO whose thickness is close to the atomic lever (0.5–3 nm), several parameters such as the hydrophilicity/thickness of GO, humidity and temperature were found to be critical for generating stable GO micropatterns. To address this issue, here, GO was functionalized with Fe_3_O_4_ nanoparticles via electrostatic interaction to synthesize hybrid magnetic nanoparticles, which could assist the transfer of GO layer to the target substrate in the presence of external magnetic force. The size of synthesized GOMNPs was found to be around 220 nm in diameter, based on the transmission electron microscopy (TEM) image and dynamic light scattering (DLS), as shown in Figure 2a,b. After the confirmation of successful synthesis of GOMNPs, patterning of GO hybrid nanoparticles was conducted.

Remarkably, as hypothesized, the GO patterns generated on the surface in conjunction with external magnetic force (MF) was superior to same patterns generated without MF, proving that MF actually facilitated transfer of GO patterns on the desired surface (Figure 2c,d). Additionally, Raman spectroscopic measurement was carried out to confirm that the pattered structure appeared on the SEM images (Figure 2(di,dii)) are the materials containing GO. As shown in Figure 2e, based on the D and G peaks of Raman spectra, clear line pattern image was observed that was perfectly matched with the SEM image, indicating that the patterned materials actually contain GO in their structure. Moreover, the magnetic force-driven GO hybrid patterns were less sensitive to environmental factors such as temperature and humidity as opposed to conventional MCP method and also, were highly stable over long period of time, proving that this newly developed GO patterning method could be useful for various areas wherein GO patterns could serve as a core electronic/optical/biomedical components.

### 2.2. Guided Synaptogenesis of Human Neuronal Cells (SH-SY5Y) on MF-Driven GO Hybrid Patterns

**Figure 3:**
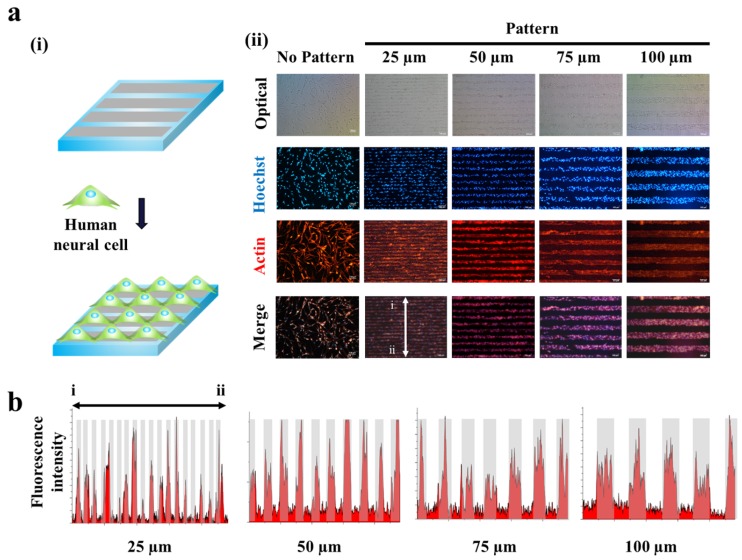
Human neuronal cells (SH-SY5Y) cultured on GO hybrid line patterns. (**a**) Schematic diagram of cells aligned on GO hybrid line patterns (**i**). Phase contrast (top row) and Fluorescence image of SH-SY5Y cells stained for nucleus (blue, second row), actin (red, third row) and merged (last row). Cells cultured on the GO hybrid pattern shows morphology of cells that followed the geometry of the GO hybrid line pattern compared to no pattern. Scale bar = 100 μm (**ii**). (**b**) Fluorescence intensity profile of phalloidin that shows the size of cell cultured on GO hybrid line patterns. (25, 50, 75, 100 μm) Red color represents the region where cells are attached, which matches the size of the pattern.

Since the magnetic force-driven GO transfer was shown to be successful to generate GO hybrid patterns with less fabrication time and high reproducibility, we next thought to investigate their potential to guide cell growth and to control cell morphologies. This is the first step to control cell alignment and to direct axonal growth/synaptic junction formation. As shown in Figure 2, human neuroblastoma (SH-SY5Y), which is a cell line that is adrenergic in phenotype but also express dopaminergic markers, were utilized as a model neuronal cell. As expected, the human neuronal cells were shown to successfully follow GO hybrid patterns with varying sizes from 25 μm to 100 μm in width. Interestingly, the widths of each cell pattern were found to perfectly match with that of GO hybrid patterns, after two days of cell culture (cell density: 100,000 cells/mL), based on the fluorescence intensity profiles achieved from the actin-stained cell images (Figure 3a,b). Since SH-SY5Y cells on GO hybrid non-patterned substrates showed neither directional alignment nor controlled cell growth, it is certain that the developed MF-driven GO hybrid patterns are not only effective to guide cell growth but are efficient to direct neural cell alignment within the range of 25 μm–100 μm.

**Figure 4:**
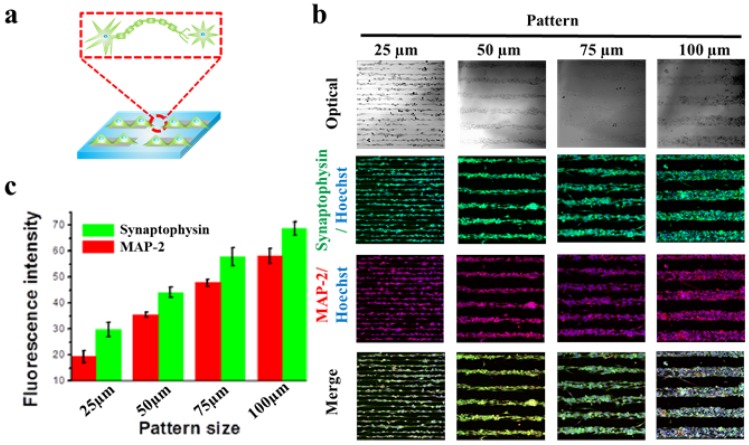
Synaptogenesis in different sized GO hybrid line patterns. (**a**) Schematic diagram of synaptogenesis of aligned cells on GO hybrid line pattern. (**b**) Phase contrast (top row) image and confocal images of aligned cells on different sized GO hybrid patterns (25, 50, 75, 100 μm) stained for synapse (green) and nucleus (blue) (second row), cytoskeleton (red) and nucleus (blue) (third row) and merged (last row) after 2 days of culture. (**c**) Quantitative comparison of synaptophysin and MAP-2 fluorescence intensity in 100 μm × 100 μm area of different sized patterns. Data are the mean ± standard deviation of three different experiments.

After the conformation of successful neuronal cell patterning, the capability of MF-driven GO hybrid patterns to control synaptogenesis of each neuron was confirmed. A microtubule-associated protein 2 (MAP-2) is a protein that is responsible for stabilizing dendritic shape during neuron development [36,37,38,39]. On the other hand, synaptophysin are a family of proteins that are known to be critical in the regulation of neurotransmitter release at synapse. It generally regulates neurotransmissions by exocytosis of synaptic vesicles that are containing neurotransmitters at any one time [45,46,47,48,49,50,51]. Hence, we used both MAP-2 and synaptophysin as the markers to confirm the neurogenesis and its corresponding synaptic formation of the patterned neuronal cells (Figure 4a). As shown in Figure 4b, both MAP-2 and synaptophysin were highly expressed in all the experimental groups, regardless of their sizes in widths. However, to investigate full potential of the MF-driven GO hybrid patterns for mimicking the human neuronal network in vitro and for utilizing them as neuroregeneration in vivo, it is important to analyze the expression level of synaptophysin and MAP-2 with varying sizes of GO patterns. As shown in Figure 4c, both synaptophysin and MAP-2 expression levels were found to increase with increase of the pattern sizes (25, 50, 75 and 100 μm). Moreover, with using 100 μm patterns, the expression of synaptophysin/MAP-2 markers were highly stable and uniformly distributed, which is critical for guided signal transduction of neurons. The mean fluorescence intensities of synaptophysin and MAP-2 of 25 μm-sized GO hybrid line pattern were 29.8 a.u. and 19.3 a.u., respectively, while 100 μm-sized GO hybrid line pattern showed 68.7 a.u. and 60.2 a.u., respectively. As a conclusion, we showed that the MF-driven GO hybrid patterns are effective for controlling behaviors/functions of neuronal cells, especially controlling both cell morphologies and synaptic junctions.

### 2.3. Synaptogenesis Guidance Due to Pattern Effect

It is certain that transplantation of neuronal cells and/or mimicking human neural network in vitro are promising candidates to treat neurological diseases/disorders. However, unlike the neuronal cells in vivo that generally show the aligned morphology toward specific direction, cells cultured in vitro show randomly distributed and non-controlled axonal growth and alignment. To address issue, Solanki et al. reported an interesting approach to guide axonal growth of differentiated neurons derived from the neural stem cells by utilizing graphene oxide-embedded nanoparticles as a cell supportive material. However, considering the fact that cell-to-cell interconnection is precisely tuned during early developmental processes to form perfect neuronal circuits (e.g., brain-to-brain, brain-to-spinal cord) and to function as a complete neuronal network, more precisely controlled neuronal cell interconnection, especially the guided synaptic junction formation, is highly preferred [52,53,54,55,56,57]. Since we already proved that our MF-driven GO hybrid patterns are effectively to guide neuronal cell growth and synaptophysin expression, we next thought to investigate whether these patterns could be utilized to mimic the synaptic junctions of the neurons growing from two different directions.

**Figure 5:**
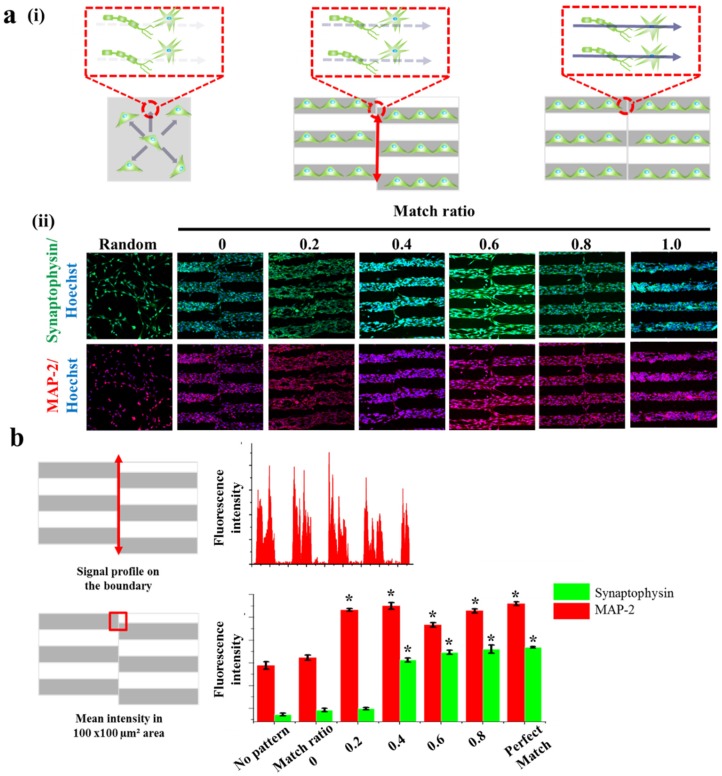
Synaptogenesis guidance due to pattern effect. (**a**) Schematic diagram of cell signal transduction in no pattern, mis-aligned GO hybrid pattern and perfectly matched GO hybrid pattern respectively (**i**). Confocal images of SH-SY5Y cells on no pattern, mis-aligned GO hybrid pattern (match ratio of 0, 0.2, 0.4, 0.6, 0.8) and perfectly matched GO hybrid pattern stained for synapse (green) and nucleus (blue) (top row), cytoskeleton (red) and nucleus (blue) (bottom row) after 2 days of culture (**ii**). (**b**) Signal profile on the boundary of mis-aligned part. Fluorescence intensity of synaptophysin is only shown on the patterned cell part. Quantitative comparison of synaptophysin and MAP-2 fluorescence intensity in 100 μm × 100 μm area of mis-aligned section. Data are the mean ± standard deviation of three different experiments. (Student’s *t*-test, *n* = 3, * *p* < 0.05).

This is important since most of the cases, especially in the spinal cord, a number of neurons form bundle-like structure named ‘nerve fiber’ to receive and transmit the signals to brain and/or to muscles. Hence, to treat spinal cord injury more efficiently, it is important to note that the disconnected part of the nerve fibers should be guided and connected to its counter parts, which is currently hard to be achieved. Based on this scientific motivation, we intentionally generated several neuronal junctions wherein neurons growing from two different direction actually meet and form synaptic junctions. By changing the match ratio from 0 (perfect mismatch) to 1 (perfect match), we hypothesized that misaligned neuronal connection, which might be occurred during the treatment of spinal cord injury, could be mimicked in vitro (Figure 5a). In order to visualize synaptophysin/MAP-2 expression at the junctions, we quantified the mean intensities of synaptophysin and MAP-2 in the certain areas that are marked as red circles in Figure 5a. Interestingly, the mean fluorescence intensity of synaptophysin expressed from the cells on normal substrate (no patterns), patterns with the match ratio of 0, 0.2, 0.4, 0.6, 0.8 and perfectly matched pattern showed 2.36, 2.46, 2.49, 3.56, 3.73, 3.8, and 3.84 a.u. respectively. Also, the mean fluorescence intensity of MAP-2 showed 3.44, 3.61, 4.66, 4.75, 4.33, 4.64 and 4.8 a.u. respectively, for same experimental groups. The mean fluorescence intensity of synaptophysin in the area wherein the neurons growing from two different directions showed highest on perfectly matched pattern and lowest on no pattern. Interestingly, unlike MAP-2 expression, synaptophysin was found to hugely increase on match ratio 0.4 and reached saturated intensity, indicating that match ratio 0.4 is critical to connect neurons and to induce stable synaptophysin expression, both of which are important for efficient signal transductions (Figure 5b). Hence, it can be concluded that the proposed various GO hybrid pattern platforms can be not only used for mimicking brain/spinal cord disconnection but also for precisely controlling axonal growth of neurons, which will ultimately be helpful for the brain/spinal cord injuries.

## 3. Materials and Methods

### 3.1. Materials

Phosphate buffered saline (PBS) was purchased from Sigma-Aldrich (Sigma Chemical Company, St. Louis, MO, USA). DMEM media, fetal bovine serum (FBS), antibiotics (penicillin and streptomycin), trypsin (0.05% trypsin, 0.53 mM EDTA-4Na) were collected from Gibco (Invitrogen, Grand Island, NE, USA). Deionized water (DIW), obtained from a millipore water system was used throughout the experiment.

### 3.2. Cell Culture

Human neuroblastoma cell line (SH-SY5Y) was purchased from ATCC (Manassas, VA, USA). The cell was cultured at 37 °C in a DMEM medium supplemented with 10% heat-activated fetal bovine serum and 1% antibiotics (penicillin and streptomycin) in a humidified atmosphere of 95% air with 5% CO_2_. The cells were grown in tissue culture-grade petri dishes. The cells were sub-cultured every 48 h of incubation at a density of 1 × 10^5^ cells/mL.

### 3.3. Fluorescence Imaging

For actin staining of SH-SY5Ys, cells were washed with DPBS (pH 7.4) two times and fixed with 4% formaldehyde solution for 10 min at room temperature (RT), then washed with DPBS for three times. 0.1% Triton X-100 in PBS were treated for 5 min, then washed with DPBS and stained with Alexa Fluor 546 Phalloidin-containing solution for 20 min at RT. Finally, to stain nucleus, Hoechst (3 μg/mL) was used for immunofluorescence imaging (Eclipse Ti-U, Nikon, Tokyo, Japan).

### 3.4. Confocal Imaging

The SH-SY5Y cells were fixed with 4% formaldehyde solution for 10 min at RT. After washing with DPBS for three times cells were permeabilized with 0.1% Triton X-100 in PBS were treated for 5 min at RT. Cells were then blocked with bovine serum albumin (BSA, Sigma-Aldrich, St. Louis, MO, USA) in DPBS for 30 min in RT to reduce non-specific protein binding. The cells were then incubated with primary antibodies 2 h in RT. The following primary antibodies were used; rabbit monoclonal (YE269) to Synaptophysin (1:200; Abcam, St. Louis, MO, USA) and mouse monoclonal (HM-2) to MAP-2 (1:200; Abcam, St. Louis, MO, USA). After the incubation of primary antibodies, the cells were washed with DPBS and incubated with secondary antibodies (FITC goat pAb to rabbit IgG (1:200; Abcam) and TexasRed goat pAb to mouse IgG (1:200; Abcam)) for 2 h at RT. The cell nuclei were also stained by Hoechst (3 μg/mL). Fluorescent images of SH-SY5Y cells were observed using a confocal laser scanning microscope. Confocal images were analyzed by Zen black. The laser power intensity for acquiring the fluorescence of synaptophysin, MAP-2 and Hoechst was kept as 90% (Max power 10 mW). In addition, the digital gain values that are controllable at detector were kept as 1, 1.25 and 1.138 for synaptophysin, MAP-2 and Hoechst, respectively.

### 3.5. Synthesis of GO-Encapsulated Magnetic Nanoparticles

Iron oxide (II, III), magnetic nanoparticles solution 10 nm average particle size, 5 mg/mL in H_2_O was purchased from Sigma-Aldrich (St. Louis, MO, USA). Single or few-layer graphene oxide was filtered centrifuging graphene oxide solution at 13,200 rpm for 30 min. Magnetic nanoparticle solution is diluted in DIW (1:10) then 0.01% of (3-Aminopropyl)triethoxysilane (APTES) was added. Then filtered Graphene oxide solution was added to finalize the reaction. The solution is reacted in Thermomixer (25 °C, 800 rpm) for 30 min.

### 3.6. Generation of GO Hybrid Patterns

By using conventional photolithographic technique, photoresist (PR) micropatterns were generated on Si wafer. Patterned wafer was then coated with (heptadecafluoro-1,1,2,2-tetrahydrocecyl) trichlorosilane for 6 h in desiccator to prevent possible damage from PDMS curing and to ease detachment of PDMS. PDMS poured on the silicon wafer is then kept in the 70 °C oven for 4 h for curing. After the PDMS stamp was detached from the silicon wafer, oxygen plasma was treated on PDMS to attract GO-encapsulated magnetic nanoparticles. GO-encapsulated magnetic nano particle solution dissolved in DIW (5.0 mg/mL) is coated on microscope slide with spin coater (500 rpm for 30 s). Coated GO-encapsulated magnetic nanoparticles were transferred on to PDMS stamp by using micro contact printing technique. Humidifier was used for increasing the level of transferring particles on to desired substrates (PDMS). After the particles are transferred on to the PDMS stamp, magnet was applied under the desired substrate (PDMS) for 1 to 10 min to increase the adhesion strength of GO-encapsulated magnetic nanoparticles on to the desired substrate (PDMS). The magnetic force was applied during the transfer of GO-magnetic particle hybrids to PDMS substrates using a permanent magnet with dimension of 1/2 × 1/8 inch (neodymium rare earth disc magnets N48) that was purchased from totalElement (Chicago, IL, USA).

### 3.7. Raman Spectroscopy

GO hybrid patterns on PDMS and SH-SY5Y cells on the pattern were investigated by Raman spectroscopy using Raman NTEGRA spectra (NT-MDT, Moscow, Russia) after 48 h of incubation, surface enhanced Raman scattering (SERS) mapping was taken by selecting 100 μm × 100 μm area with 32 × 32 data points. Raman spectra were recorded using a near-infrared (NIR) laser emitting light at a wavelength of 633 nm. Ten scans of 1 s from 1200 cm^−1^ to 1800 cm^−1^ were recorded.

## 4. Conclusions

Overall, we have developed a novel MF-driven GO hybrid pattern that were highly effective for controlling synaptogenesis. The GO hybrid pattern arrays, with different sizes, were successfully generated on flexible PDMS without any chemical linkers and complex processes. By using magnetic force, GO-encapsulated magnetic nanoparticles were effectively transferred on to the desired substrate with less amount of time and high reproducibility when compared with conventional MCP method, which was confirmed by SEM and Raman imaging. Due to the property of GO that enables long-term stable cell culture, human neuronal cells could be effectively patterned on PDMS substrates with different pattern sizes. Additionally, we found that the MAP-2 and synaptophysin expressions, which are two important indicators for neuronal cell development and synaptic junction formation, could be controlled by GO hybrid patterns. Finally, by generating several types of neural cell disconnection to mimic the process to cure damaged nerve fiber, we found that specific match ration is important to induce stable synaptophysin expression and to form synaptic junctions (match ratio = 0.4). Hence, it can be concluded that the proposed method, MF-driven GO hybrid patterns, could be highly useful for modeling of brain diseases and spinal cord injuries.
